# The State-of-the-Art Overview to Application of Deep Learning in Accurate Protein Design and Structure Prediction

**DOI:** 10.1007/s41061-024-00469-6

**Published:** 2024-07-04

**Authors:** Saber Saharkhiz, Mehrnaz Mostafavi, Amin Birashk, Shiva Karimian, Shayan Khalilollah, Sohrab Jaferian, Yalda Yazdani, Iraj Alipourfard, Yun Suk Huh, Marzieh Ramezani Farani, Reza Akhavan-Sigari

**Affiliations:** 1 Division of Neuroscience, Department of Cellular and Molecular Medicine, Faculty of Medicine, University of Ottawa, Ottawa, ON Canada; 2 Faculty of Allied Medicine, Shahid Beheshti University of Medical Sciences, Tehran, Iran; 3 Department of Computer Science, The University of Texas at Dallas, Richardson, TX USA; 4 Electrical and Computer Research Center, Sanandaj Azad University, Sanandaj, Iran; 5 Department of Neurosurgery, Faculty of Medicine, Tehran Medical Sciences, Islamic Azad University, Tehran, Iran; 6 Goergen Institute for Data Science, University of Rochester, Rochester, NY USA; 7 Immunology Research Center, Tabriz University of Medical Sciences, Tabriz, Iran; 8 Institute of Physical Chemistry, Polish Academy of Sciences, Marcina Kasprzaka 44/52, 01-224 Warsaw, Poland; 9 Department of Biological Engineering, Inha University, Incheon, Republic of Korea; 10 Department of Neurosurgery, University Medical Center, Tuebingen, Germany

**Keywords:** Deep learning, Protein structure prediction, Rational design, Protein engineering

## Abstract

In recent years, there has been a notable increase in the scientific community's interest in rational protein design. The prospect of designing an amino acid sequence that can reliably fold into a desired three-dimensional structure and exhibit the intended function is captivating. However, a major challenge in this endeavor lies in accurately predicting the resulting protein structure. The exponential growth of protein databases has fueled the advancement of the field, while newly developed algorithms have pushed the boundaries of what was previously achievable in structure prediction. In particular, using deep learning methods instead of brute force approaches has emerged as a faster and more accurate strategy. These deep-learning techniques leverage the vast amount of data available in protein databases to extract meaningful patterns and predict protein structures with improved precision. In this article, we explore the recent developments in the field of protein structure prediction. We delve into the newly developed methods that leverage deep learning approaches, highlighting their significance and potential for advancing our understanding of protein design.

## Introduction

Proteins are prerequisite components of living organisms whose functions are dictated by their three-dimensional structures, which in turn are predominantly bound to their amino acids sequences [2]. At the time of writing this article, structures of more than 177,000 proteins have been submitted to publically accessible online databases, resulting from meticulous experimental research directed at determining three-dimensional structures using, for example, crystallography and neutron magnetic resonance (NMR) (Table 1). Such experiments are time-consuming, and more feasible options than those determining the structures of hypothesized proteins and synthesized peptides according only to their amino acid sequences have been developed that are more rapid and less time-consuming. More complications will arise when atomic accuracy without homologous counterparts is needed [3]. Only a few methods have attempted to tackle this specific issue; these have used different approaches but aspired to achieve the same goal: the atomic accuracy of an angstrom [4].

**Table 1:** Experimentally validated methods for solving protein structure in the Protein Data Bank database according to the method applied

PDB data distribution by experimental method (proteins only)	Count
X-Ray	155,029
NMR	12,190
EM	10,262
Multiple methods	191
Neutron	72
Other	32
Total	**177,776**

At the time of writing this article, X-ray crystallography remains the predominant approach for protein modeling

*EM* Electron microscopy,* NMR* neutron magnetic resonance,* PDB* Protein Data Bank database

To bridge the gap between amino acid sequence and a protein structure that governs functionality, the scientific community of computational biology should face the problem of developing a method for accurate prediction of protein structure [5]. There are predominantly two logical schemes, one focusing on evolutionary homology and the other focusing on the physical interactions of thermodynamics and kinetics. Focusing solely on the latter has proven increasingly challenging due to the complexity and intractability of many variables responsible for protein stability. The former method employs homology to solve and predict protein structure according to evolutionary history [6]. In recent years, deep learning techniques (See Glossary at end of article) have been employed to interpret the correlation between experimentally solved protein structures and their corresponding amino acid sequences. Due to the large quantity of data available in both types of databases, there remains much to be desired when dealing with predictions involving structures with no experimentally solved homolog counterparts [7]. The additional information needed to overcome this obstacle can be obtained through the process of mining co-evolutionary indicators in the alignment of multiple sequences. However, even with the aid of a deep learning neural network, it has not yet been possible to predict the interaction between residues that ultimately assemble into a protein structure. Additionally, in recent years, an inverse dilemma has attracted the attention of the biotechnology community, namely the construction of an amino acid sequence that folds into a specific three-dimensional structure. This would serve as a logical and rational design for a specific interaction [8]. Protein modeling algorithms are fundamentally demanding tasks. Recent advancements in computational hardware, including both central processing units (CPUs) and graphics processing units (GPUs), along with the rapid progress in algorithm development, are facilitating significant strides toward attaining meaningful solutions in protein structure prediction.

In this review, our aim is to describe the highlights of these breakthroughs in protein modeling with a focus on deep-learning methods. It is an exciting new vantage point worth exploring that is pioneering the rational design approach.

## Multi-Stages Modeling

The prediction of protein structures is rooted in Anfinsen’s hypothesis, formulated in 1961, which posits that the primary sequence of amino acids alone encodes the necessary information for precise folding. This hypothesis suggests that the native state of a polypeptide possesses the lowest free energy and can be envisioned as an energy landscape. Subsequent refolding experiments have provided empirical evidence supporting this hypothesis, now widely recognized as Anfinsen’s dogma [9]. The main challenge to this approach was first identified by Levinthal, who described it as a conceptual roadblock, taking into account that each amino acid has a small finite number of possible backbone states and, as the chain grows, the total size of the searchable alternative conformations become unattainable [see 10]. To circumvent this roadblock, it is crucial to recognize that the landscape of the energy is not, in fact, a flat plane with a single sink-hole but rather an overall funnel shape that directs sampling toward the native conformation; accepting this perception eliminates the necessity of exploring the entire conformational space. In short stretches, local interaction between amino acids can direct the sampling toward the near-native conformation [11]. In globular proteins, hydrophobic residues are predominantly buried in the core of the protein, increasing the van der Waals interactions while restricting the formation of entropically unfavorable grooves in the water solution. Also, the bias toward a strong backbone limits the number of flexible positions each side chain can assume in the proximity of a specific set of rotamers [12]. As a protein folds to its natural state, inevitably, some polar groups are buried in the core and cannot contribute to the total stability of the native state; consequently, this potential decrease in stability is compensated for through the formation of hydrogen bonds and disulfide bridges. This concept can be utilized to distinguish between native states and other compact ones. As hydrogen bonding in the backbone and hydrophobic burial of residues in the core are detectable in low-resolution modeling systems, specific modeling of side-chain flexibility requires a more computationally intensive analysis [13].

**Fig. 1:**
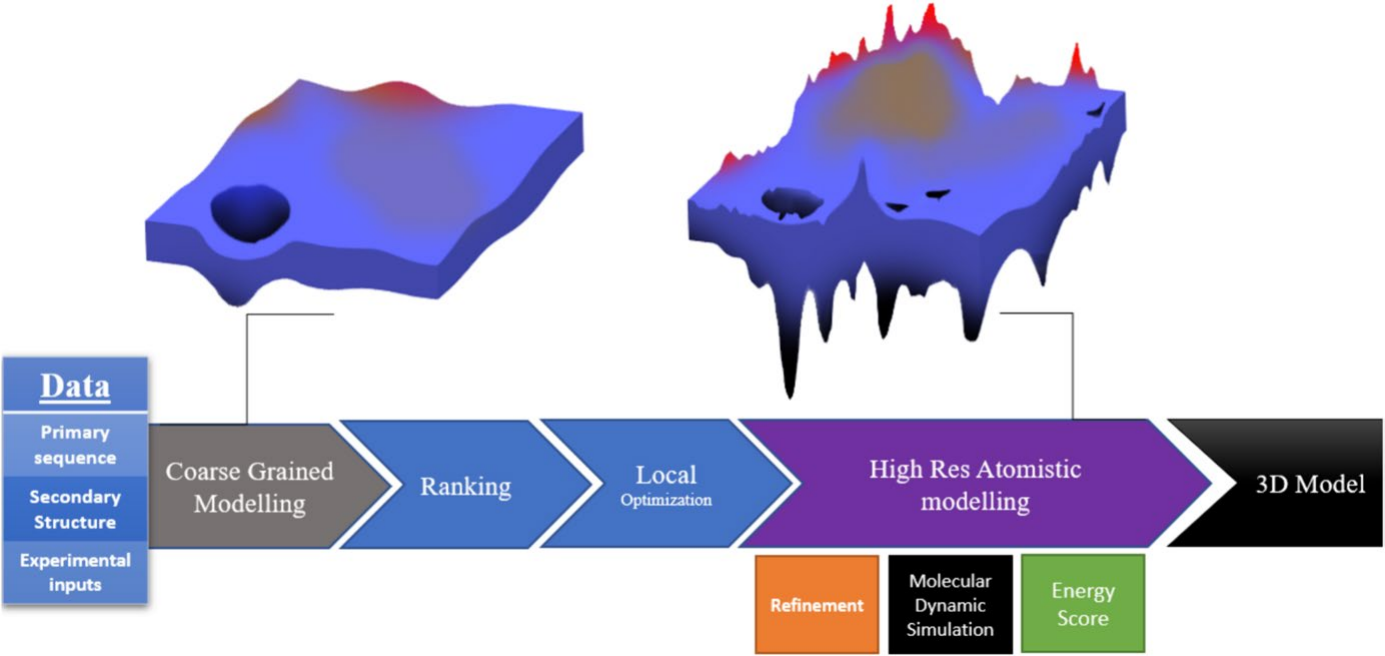
Following the coarse-grained modeling stage, the focus shifts to the higher resolution atomistic level. Here, the modeling process becomes more intricate, incorporating the modeling of flexible side chains and protein structure refinement.* 3D* Three-dimensional,* Res* resolution

Modeling approaches for protein structure prediction are typically categorized into multiple levels with varying resolutions. The sampling of hydrophobic interactions, formation of secondary structure, elimination of overlaps and overall backbone conformation are addressed using a coarse-grained energy function [3]. This allows for faster exploration of the conformational space. Subsequently, at the higher resolution of the atomistic level, the focus shifts toward modeling flexible side chains and refining the structure. This stage takes into account multiple local minima in the energy landscape. Due to the increased level of detail, this refinement step is more computationally intensive [14]. (Fig. 1).

In previous research, the prediction of protein models with the aid of known related proteins, known as template-based modeling, was fundamentally distinct from the prediction of proteins without any known counterparts. The choice depended on the availability of known structures in the databases; as such, the former method employed alignment of a known protein with the homology in mind, while the latter method focused on the energy function and sampling from alternative forms. Although template-based modeling can often generate accurate predictions, it must provide a measurable understanding of the physical principle of the folded structure [4]. Recently, however, the distinction between the two methods has become clearer as the template-based methods integrate energy refinement. At the same time, the novel fold modeling system incorporates deep learning to sample the information currently available in protein databases. This method can recognize the similarity between small fragments of polypeptides and identify the least common denominator when assimilating the backbone and side chains during multiple levels of refinement.

## Template-based modeling

In a typical template-based modeling scenario, a preferable structure closely related to the query must be selected initially. This selection can consist of a simple BLAST (Basic Local Alignment Search Tool) search for a single sequence or multiple-sequence alignment from different proteins of known structures. Following the selection of a suitable backbone, modifications, such as deletion, insertion and mutation in the query, will be accounted for during the optimization of side chains. The initial backbone will be rotated and reconstructed solely around those specific modifications, and then the final model will be constructed. For the less closely related targets, more sophisticated methods, such as fragment-based modeling, will be utilized; however, when homology in the targets with the aligned results of < 20% is achieved, the accuracy of template-based modeling will plummet and render it unsuitable for gaining reliable results[15]. Approximately 60% of known protein families can accommodate template-based modeling to provide meaningful data regarding three-dimensional structures.

## Template-free Modeling

In contrast to template-based modeling, the global similarity is absent in the scenario of template-free modeling. Thus, a sampling scheme for generating candidate structures followed by a ranking criterion would be employed. First, multiple alignments are used to predict local features, such as torsion angles of the main backbone and different secondary structures. Non-local features, such as the distances between residues and clashes, are also calculated, following which, after refinement, ranking between candidates will be utilized for the final prediction. During the initial sampling, usually short fragments of 3–12 residues in length are taken from experimentally modeled structures [16]. Fragment sampling relies not only on sequence similarity but also on the secondary structures and torsion angles. At this stage, the prediction of a protein is usually guided by the random brute forcing of employing the Monte Carlo simulation. This simulation randomly substitutes fragments of 25 residues in length in a small backbone, calculates the energy and compares this energy with another randomly selected fragment. If the energy is lowered after the insertion, the move is favored; in the case of an increase in the energy, the probability of a suitable insertion is dramatically lowered by the criterion of Metropolis [4]. Thousands of simulations are usually needed to find the lowest state of energy. However, to some extent, the coarse-grained function can mitigate the complexity of such simulations for a faster result. In the absence of global similarity, fragment-based modeling offers some advantages, such as using experimentally curated fragments to ensure that the final model has the local protein features without solely relying on meticulous energy calculation. Monte Carlo simulation is efficient for small domains with predominant alpha-helical structures. In contrast, long-distance residue contacts in beta-strands and topologically complex domains pose the biggest challenge for this simulation. Real-world applications, such as drug design and inverse approaches for protein design, rely on finer features in the near-native state of a protein, such as side-chain packing. Therefore, the coarse-grained energy function in fragment assembly is unable to fill the gaps in required details; hence, many refinement strategies are proposed to achieve a detailed model. Refinement strategies, such as molecular dynamics, incorporate precise energy functions in conjunction with exploring the surrounding space. In molecular dynamic simulation, the surrounding space in a hypothetical box is filled with water molecules, and the simulation of the trajectory of the target molecule is calculated in minuscule time-frames according to Newton’s Law of motion and energy [17]. Recently, this refinement strategy has been further enhanced by incorporating energy minimization and the sampling of side-chain rotamers, demonstrated by the Rosetta tool [18].

## Machine Learning-based Methods

More sophisticated methods have emerged with the growing demand for more precision in modeling and in defining the more disguised features like residue-residue contacts. In earlier studies, machine learning models were employed to predict features like torsion angle and accessible residues. More recently, predictions like the inter-residue distances have paved the way for deep-learning techniques to be able to familiarize themselves with patterns in available sequences in databases and their correlated structures. Advances in image analysis have facilitated the prediction of contact maps [19]. Methods like DeepContact use deep learning to accommodate themselves with large amounts of data of known proteins and their contact maps and alignment to recognize spatial contacts [20]. Deep learning is also contributing to a recently emerging field of protein design that, by nature, is an inverse dilemma to an already existing protein prediction challenge. In the field of protein design, finding the conformation with the lowest energy is not the priority; rather, the aim is to determine a specific sequence that folds into a stable desirable conformation or incorporates itself into a binding interaction. Yet the toolsets needed for such approaches are very similar to previous prediction attempts [21] and, similar to protein modeling, template-based designs and de novo designs are employed. In template-based protein design, existing proteins are modified to gain new functions, while in the de novo designs approach, desirable interactions and physiochemical restrictions dictate the desirable backbone and side chains. In contrast to repurposing naturally occurring proteins, the de novo designs method allows for the creation of new structures without the reliance on evolutionary restrictions. It is worth mentioning that the energy function is also used to rank the possible conformations with favorable side chains and interactions in both de novo and template-based protein design [22].

 The first step in the de novo approach in protein design is the assignment of a particular fold or a topology. The fold is then used to induce a particular function. The number of conformations for a hypothetical fold can be extensive, but only a few of them are thermodynamically stable and properly packed. Similar to structure prediction, small fragments of naturally occurring folds are often used in conjunction with specific restrictions in residue distance. This strategy has been used to design many protein folds that are not limited to helical conformations, such as the folds that solely consist of beta-sheets and turns. This approach has also successfully been used to make alternate folds from naturally occurring helix-turn-helix motifs to form pockets with active site functionality [23]. In the case of coiled proteins with a limited number of alpha helix fragments, it is also possible to use the purely parametric design, initially with a limited number of parameters, for obtaining the desirable backbone in a systemic manner [24]. Certain spatial parameters allow for the tight packing of coiled folds with stronger interactions. In de novo protein design, the usual goal is either the correct protein–protein interaction or the protein–ligand interaction; in both cases, a sufficient hydrophobic packing between two molecules is desirable. Hence, the natural next step is docking [25]. Protein–ligand docking is feasible with many toolsets, chief among them are the Molegro virtual docker [26] and the accelerated Vina–GPU docker, both of which yield suitable results for small ligands [27]. On the other hand, protein–protein docking is more computationally intensive. Previously, the ZDOCK tool was used as the benchmark for protein–protein docking; the recently developed Graph Neural network (GNN-Dove) [28] utilizes a deep learning approach to extract the interface area and has proven to be a more robust tool compared to previous methods.

 In protein design, regardless of the initial method, which can be template-based or de novo, some optimizations are employed since a particular amino acid sequence is involved in the binding or stabilization of the final protein [29]. Sequence optimization software incorporates energy functions to account for the favorability of binding or stabilizing sequences while searching for the most suited alternative sequences. Similar to structure prediction approaches, the same energy function is used during the design and refinement steps. In this context, deep learning is also utilized to train the energy function to predict the experimentally proven changes in energy corresponding with shifting different chemical groups. Multiple methods, such as genetic algorithms and simulated annealing, have also been developed to identify better substitute sequences. Rosetta software uses simulated annealing to identify rotamers with lower energy. Despite the rapidity of this method, it does not guarantee that the lowest possible energy will be found. A vulnerable area in protein design is the sensitiveness of the calculations needed to determine even small changes in the conformation of the backbone, and since the energy calculation is based on all the atoms in the protein model and cannot account for all the variables that contribute to the energy in a protein, this can pose a challenge. To address this issue, TERtiary Motifs (TERMs) or tertiary structural motifs that are derived fragments of residues from known proteins are first introduced in the Protein Data Bank (PDB) database. TERMs are then used for optimizations that share similar energy functions [30].

## Application of Rational Protein Design

During the last decade, applications of protein design in research, medicine and industry have grown exponentially. The premise of the rapid creation of new proteins in conjunction with the development of new computational algorithms have led to researchers suggesting multiple solutions to their problems in quick succession. Creating specific mutations for more thermally stable proteins with higher expression is manageable using the multiple alignments in conjunction with the Rosetta simulation. For example, this approach can increase the thermal tolerance of proteins, such as the RH5 protein involved in malaria invasion [31]. In medicine, the ability to change the properties of binding specificity is a powerful tool to manipulate cell signaling pathways. Another example of novel assemblies using simulations was demonstrated in designing dual-specific antibodies by self-assembling two unique light chains. This resulted in each arm recognizing different targets and has been proven to be useful in recruiting immune T cells to a specific target [32]. Another example of de novo protein design is the creation of interleukin (IL) mimics that confer anti-cancer properties by binding to their receptors without inducing toxicity [33]. One of the main strengths of protein design can be seen in self-assembling nanocages. These symmetrical oligomers are constructed using homodimers and homotrimers as building blocks and designed with complementary interfaces for stable assembly. Similar to viral capsids, nanocages can pack RNA molecules and have many applications in nanoparticle vaccines [34]. Regulating signaling pathways is an intriguing concept for de novo protein design and, in particular, can be utilized in inflammatory pathways [35]. This concept incorporates a one-sided interface design in which one of the proteins in an interaction is mutated. Since excessive increases in polar residues in the mutated protein can be detrimental to stability and proper folding, more hydrophobic patches on the target surface are taken advantage of during the design process. Protein–protein interaction can further be improved by means of scaffolding [36], which encompasses the binding motif in a properly folded scaffold. Previous attempts consisted of sampling the fragments of receptor-binding helices of IL-2 that were integrated into a de novo-designed protein. The resulting molecule conferred the anti-cancer properties in colon cancer mouse models while inducing less toxicity. Deep-learning mediated protein design is also useful when immune regulatory and anti-inflammatory properties are desired; this goal is achievable by sampling and adding the mutations in motifs that are deemed favorable substrates for major histocompatibility complexes in target molecules [37]. A new perspective in de novo protein design is to create multiple protein structures that are computationally sampled for suitable binding pockets. In a previous study, multiple beta barrels were computationally designed in a manner to enhance the fluorescent emission for localization imaging inside the cell. Accuracy is one of the main challenges when computational methods in de novo protein design are employed in catalytic scenarios. The typical 1-angstrom accuracy of computational methods cannot fully account for the sub-angstrom nature of transitional states of binding to substrates and the subsequent release of the products in enzymes. Although additional side chain design in surrounding residues is usually performed for the stability of the protein, the naturally evolved enzymes incorporate many different mechanisms, such as entering the substrate and releasing the product in quick succession, which is currently challenging for available methods to evaluate. Many important proteins, like those involved in signal transduction pathways, have more than one conformation. Multiple conformations enable the protein to switch between alternate forms. A multi-state protein design has been used to incorporate different time scales in the conformational transition of a protein that is functionally relevant and is at low energy states for different conformations that a backbone can accommodate [38]. These flexible cases are often calculated by the combination of molecular trajectory simulation and the analysis of the energy landscape.

### Limitations of Rational Protein Design in Functional Dynamics

The rational methods used in protein design offer valuable insights into protein structure and function; however, they are not without limitations, including those mentioned below.

#### Inability to Capture Complex Functional Dynamics

One of the major limitations in rational designing is the inability of current methods to accurately capture the intricate dynamics of protein function. Proteins often exhibit complex conformational changes and dynamic motions that are essential for their biological activity. Rational design methods struggle to account for these dynamic behaviors adequately.

#### Dependence on Simplified Models

Rational protein design often relies on simplified models of protein structure and function. These models may overlook important factors, such as solvent effects, ligand interactions, and allosteric regulation, leading to inaccuracies in predictions.

#### Challenges in Predicting Protein Flexibility

Predicting the flexibility of proteins, especially in response to environmental changes or binding events, remains a significant challenge for rational design methods. Flexibility is crucial for protein function, yet accurately predicting and manipulating flexibility a computationally daunting task.

These limitations significantly impact protein engineering efforts, which can lead to engineered proteins with altered dynamic behaviors.

## Task-specific tools

As the trend of protein design moves toward the semi-rational approach, employing direct evolution, which relies on high-throughput systems and precise simulation of rational designs in unison, is inevitable. To fulfill this task, a myriad of tools have been developed during the last decade, which are becoming continuously more sophisticated. On the bright side, many of those tools focus on distinct tasks and can be utilized for many particular cases by offering many solutions to a single problem. The negative implication is the overwhelming number of these toolsets that, in many cases, could be more user-friendly for practitioners [1]. The majority of these tools rely on multiple-sequence alignment to gather information about allosteric networks, transport pathways, and binding sites. We have gathered a list of these tools that may be less known but offer applicable approaches to tackle these challenges (Table 2).

**Table 2:** A list of more task-specific tools

Program/toolset	Application	Link
PPI3D ISEE MutaBind DisruPPI	Protein–protein interaction	http://bioinformatics.ibt.lt/ppi3d https://github.com/haddocking/iSee http://www.ncbi.nlm.nih.gov/projects/mutabind
CaverDoCK	Ligand transport	https://loschmidt.chemi.muni.cz/caverweb
PDB2Graph AlloSigma STRESS	Allostery	http://bioinf.modares.ac.ir/software/pdb2graph http://allosigma.bii.a-star.edu.sg https://github.com/gersteinlab/STRESS
BioStructMap	Data integration	https://biostructmap.burnet.edu.au
DynaMut	Dynamic simulation	http://biosig.unimelb.edu.au/dynamut/

## RoseTTAFold and AlphaFold2

As discussed in the above text, many attempts have been made during the past decade to solve the problem of protein modeling, whether used for prediction or for design purposes. Until recently, most developments revolved around energy function and force fields to account for interactions in atomic levels [39]. Realistically, the myriad of parameters cannot be collectively realized in such simulations, and the accuracy of predictions based on the dynamics simulation is bottlenecked by the embedded physics of the force field. Also, molecular dynamic simulations are usually performed on a time scale of < 1 ms; while it can accurately account for smaller molecules, this time scale renders this method unsuitable for larger complexes. Popular Monte Carlo-based methods [21], such as Rosetta, were developed to identify the lowest energy state; hence, their usefulness declines when used for larger molecules. Millions of parameters must be considered to comprehend and utilize the relation between amino acid sequence and structure.

 Very recently, deep learning-derived methods, such as AlphaFold and RoseTTAFold, have reached desirable accuracy when dealing with larger complexes [40]. Unlike previous methods, these new methods make no assumptions about the form or function of atomic interaction; rather, the training of the network is done directly on thousands of already experimentally determined structures. Unlike molecular dynamic simulations, the trajectory is directly optimized by deep learning training in a series of updates; hence, the challenge of global optimization in large spaces is overcome. Although these methods are developed by training the network with extracted structural information from already determined proteins, they can predict the structure from the amino acid sequence without relying on evolutionary information. This indicates a rich understanding of these methods about sequence-structure relationships [41]. Very recently, RoseTTAFold was trained to recover sequences from the structure. The application for this accomplishment is to enhance the quality of limited experimental data by completing the missing gaps in the structure. Since RoseTTAFold and AlphaFold use different network training and parameters, combining both in predicting structures and even more ambitious goals, such as full proteome interactions, has proven to be more accurate than using either individually [42].

**Fig. 2:**
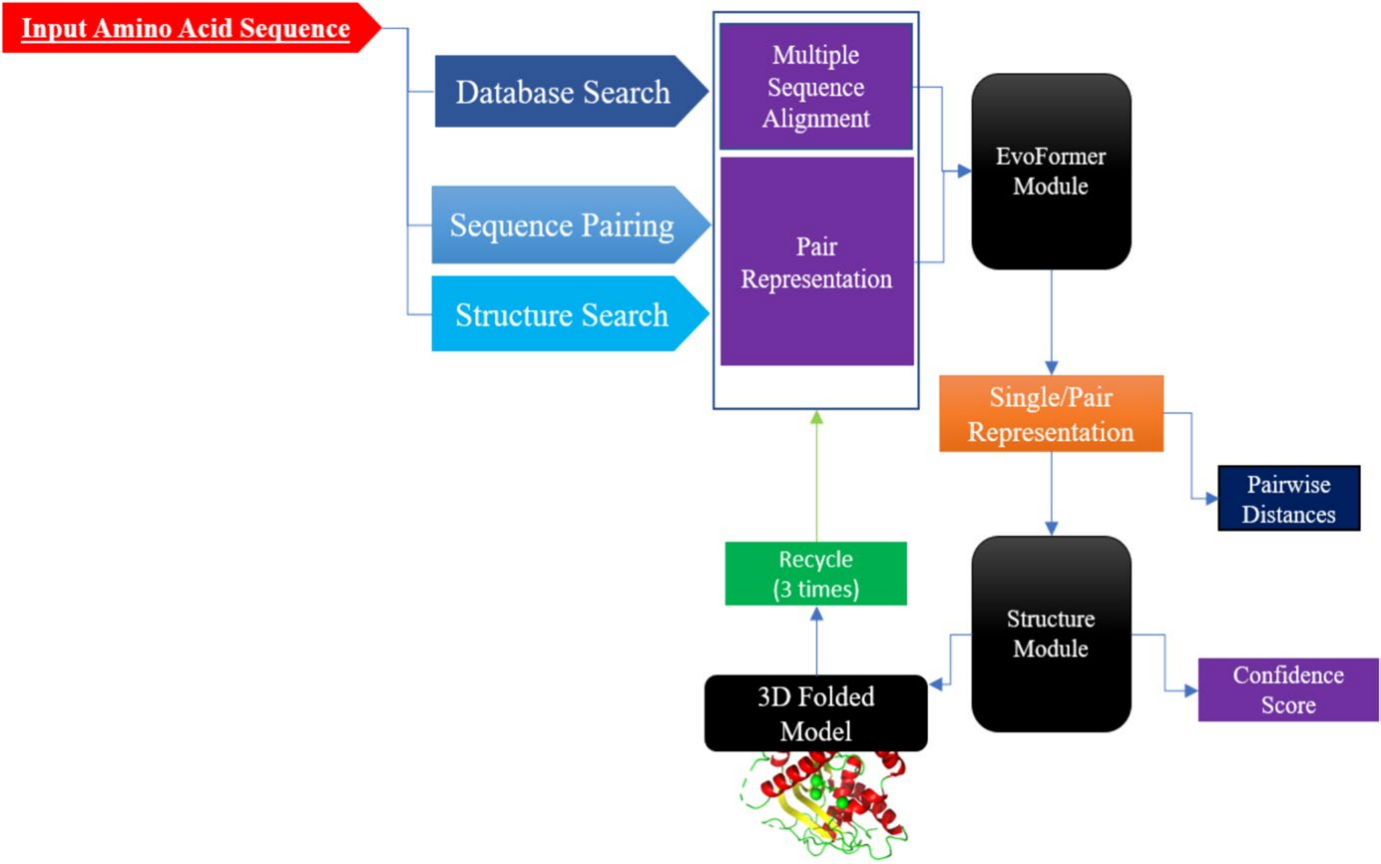
AlphaFold2 workflow works on the basis of pair representation. It refines the representations for both the multiple sequence alignment and the pair interactions but also iteratively exchanges information between them

The gold standard for the accuracy and performance in the structure prediction of newly developed approaches is the CASP (Critical Assessment of protein Structure Prediction), which occurs once every 2 years with a simple yet robust method by using the recently solved structures that have not been publicly disclosed and thus considered to be blind prediction tests for competing methods [43]. During the CASP14 assessment in July 2020, AlphaFold2 achieved tremendously more accurate results for structure prediction with a median backbone accuracy of root mean square deviation (RMSD) of 0.9 Angstrom (Å) at 95% residue coverage; a RMSD of 2.8 Å was previously considered the record of other competing methods [44]. By training a neural network with data based upon physical, evolutionary homologous structures and geometric constraints of thousands of determined PDB structures, AlphaFold2 was able to predict the structure of > 350,000 unique sequences from UniClust30 (Fig. 2) [45]. AlphaFold2 uses geometric inductive bias to render structures that are trained from PDB; by doing so, it eliminates the requirement for handcrafted features, such as the score function for hydrogen bonding. This allows AlphaFold2 to fill in the missing experimental data and to produce accurate results in challenging cases, such as structures with intertwined homomers [46, 47]. Instead of the CPU, AlphaFold2 leverages the graphic processor unit. Currently, it uses roughly 1 GPU–minute per 384 residues. A parallel development in high-end processors, particularly in GPUs, drove applications to be more lenient toward GPU CUDA-Cores instead of CPU for such tasks. Although AlphaFold2 can run locally on a CPU, it benefits greatly from stronger GPUs with a high amount of vRAM. This application is publicly available in GitHub and can be run locally with a database of around 2TBs. However, in collaboration with Google, it can also be utilized as a user-friendly online tool with a decent performance in collab.research.google.com [48].

## Concluding remarks

During the last 2 years, PDB has integrated computed structure models (CSM) in its database and search algorithm. At the time of writing this review, more than 1 million CSM entries had been registered in PDB and were publicly available. This shows that alongside 200,000 experimentally determined structures[49], CSM models have been accepted by the research community and are leveraged as an invaluable tool that facilitates a multitude of previously challenging aspects of de novo protein design and subsequent modeling. The extent of transformative progress in the field of computational biology during the past year has been astonishing. To train a network with a myriad of data and account for astronomically more parameters than any conventional method promises an exciting future where the accuracy of the prediction of the progress in this field is far behind the accuracy of predicting protein structures.

## Data Availability

Not applicable.

## Glossary

Deep Learning: Deep learning is a subfield of machine learning that focuses on training artificial neural networks to perform complex tasks. It involves the use of multiple layers of interconnected nodes or neurons.
Molecular Dynamics Simulation: A computational technique that simulates the movement and interactions of atoms within a protein structure over time. Molecular dynamics simulations can refine protein models by exploring dynamic behaviors, flexibility, and interactions with surrounding molecules.
Nuclear Magnetic Resonance: This is a powerful analytical technique used to study the properties of atomic nuclei within a magnetic field.
Refinement: The process of iteratively improving the accuracy and reliability of predictions related to protein structure, function, interactions, and other properties.
Rotamer: A rotamer refers to one of the possible conformations or spatial arrangements that a molecule can adopt due to the rotation of specific bonds.
